# Pattern of cigarette smoking: intensity, cessation, and age of beginning: evidence from a cohort study in West of Iran

**DOI:** 10.1186/s13011-020-00324-z

**Published:** 2020-10-27

**Authors:** Behrooz Hamzeh, Vahid Farnia, Mehdi Moradinazar, Yahya Pasdar, Ebrahim Shakiba, Farid Najafi, Mostafa Alikhani

**Affiliations:** 1 Research Center for Environmental Determinants of Health, Kermanshah University of Medical Sciences, Kermanshah, Iran; 2 Substance Abuse Prevention Research Center, Health Institute, Kermanshah University of Medical Sciences, Kermanshah, Iran; 3 Behavioral Disease Research Center, Kermanshah University of Medical Sciences, Kermanshah, Iran; 4 Nutritional Sciences Department, School of Public Health Kermanshah University of Medical Sciences, Kermanshah, Iran; 5 Social Development and Health Promotion Research Center, Kermanshah University of Medical Sciences, Kermanshah, Iran; 6 Research Center for Environmental Determinants of Health, School of Public Health, Kermanshah University of Medical Sciences, Kermanshah, Iran

**Keywords:** Cigarette smoking, Prevalence, Smoking cessation

## Abstract

**Background:**

Smoking is a social epidemic and one of the main risk factors for premature deaths and disabilities worldwide. In the present study, we investigated the Pattern of Cigarette Smoking: intensity, cessation, and age of the beginning.

**Methods:**

Data collected from the recruitment phase of Ravansar (a Kurd region in western Iran) Non-Communicable Disease (RaNCD) cohort study was analyzed by using Chi-square test, univariate and multivariate logistic regressions, Poisson regression, and linear regression.

**Results:**

Totally 10,035 individuals (47.42% males) participated in the study. Mean age was lower for males (47.45 yr) than for females (48.36 yr). Prevalence of smoking was 20% (36.4% of males and 5.23% of females). Compared to female participants, males showed a 7-fold higher prevalence of smoking and started smoking about 4 years earlier. Being married, having a lower BMI, living in rural areas, and being exposed to secondhand smoke (SHS) were predictors of higher smoking prevalence rates. Furthermore, current exposure to SHS, higher smoking intensity, later smoking initiation, male gender, younger age, lower education, and lower BMI were related to lower likelihood of stopping smoking. Heavy smokers began to smoke about 4 years earlier than casual smokers did. Finally, being divorced/ widow/ widower/ single and childhood exposure to SHS were found to increase the likelihood of becoming a smoker.

**Conclusions:**

Based on present research results, health programs specific to smoking cessation should take socio-demographic factors, smoking history, and current smoking behavior into account.

## Background

Cigarette is a rubbish substance readily available to the general public and smoking is highly indecent socially. Young people and teenagers turn easily to smoking and become addicted to cigarettes because they are inexpensive and abundant [1]. Smoking is one of the leading causes of preventable diseases and deaths, being responsible for approximately 6 million deaths per year worldwide [2]. Smoking creates higher mortality rates in the population consisting of men and women starting to smoke between adolescence and middle age in addition to imposing heavy financial costs. Since the 1960s, regular male/ female smokers have shown the age-adjusted relative risks of twice/ trice higher than those shown by non-smokers. Smoking shortens life expectancy by 10 years on average because tobacco use puts people at high risk of developing related diseases, with people sometimes dying from common health problems such as pneumonia and/or from surgical operations involved [3].

Smoking is associated with increased medical costs, reduced life expectancy, aggression, crime and theft. Obviously, proven relationships exist between smoking and respiratory, cardiovascular and cancer diseases [4, 5].

Although many adverse health effects of tobacco occur later in life, smoking can be associated with educational problems [6, 7], consuming other kinds of substances [8], delinquency [9], damaged psycho-social functions [9], depression and anxiety [10], and high-risk sexual behaviors [9]. Although prevalence rates of smoking began to decline in most countries during recent decades, it is still high in many parts of the globe.

Iranian tobacco controlling policies have not been implemented effectively [11]. According to data available on the 18–65-year-of-old age group, prevalence of cigarette smoking was 25.4% in Iran in 2011 [12].

Average estimates from the National Bureau of Statistics indicate an annual consumption of 55 billion cigarettes in Iran. Based on international sources, 2–3 times higher than the money paid for purchasing cigarette is spent on medical and health expenditures in our country. As indicated by reports from World Health Organization (WHO), if status quo of tobacco use remains unchanged, Iran will be among regional countries with the highest increase in tobacco consumption by the next 40 years [11].

As smoking accounts for 50% of premature deaths, healthcare system gives the priority to smoking cessation [13]. It is difficult to stop smoking successfully, with its relapse being very common [14]. Many smokers make several attempts to quit cigarettes, but they usually fail to keep abstinence [15]. In 2018, 55.1% (21.5 million) of adult smokers self-reported that they had made an attempt to quit smoking in the past year, only 7.5% (2.09 million) of whom stopped smoking successfully [16]. A study conducted in Iran indicated that only 2.7% of smokers were able to quit cigarettes [17]. Smoking cessation depends on many factors capable of increasing the likelihood of success in quitting cigarettes. Recognizing and focusing on these key factors will help to avoid costs of treatment imposed by smokers on national healthcare system in the future, rising the levels of public health [18]. Therefore, it is very important to find factors related to the success of the smoking cessation [19].

For this reason, it is important to have knowledge of socio-demographic differences, type of smoking habits, age of smoking initiation, smoking intensity, and ability to quit cigarettes in any population.

Given the relatively high prevalence of smoking in Iran and harmful role it plays in causing various diseases and in increasing mortality rates, present study was conducted to identify the pattern of Cigarette Smoking: intensity, cessation, and age of beginning.

## Methods

### Design and sample

Ravansar Non-Communicable Disease (RaNCD) cohort study is a part of PERSIAN (Prospective Epidemiological Research Studies in Iran) Cohort, focusing on Ravansar permanent residents in the 35–65-year-of-old age group. All 19 cohort sites (Covering Iranian people from different ethnicities) of PERSIAN used the same questionnaire containing different parts. Details of the study design and rationale for conducting PERSIAN cohort were discussed elsewhere [20].

Geographically located in west of Iran, Ravansar is a town in Kermanshah province close to Iraqi borders. Most residents belong to Kurdish ethnicity. Recruitment phase of the study started in November 2014 and ended in February 2017, during which more than 10,000 people signed the letter of informed consent to participate in the study [20].

To collect information, participants were invited to the study site. After the enrolment of participants, bioelectric impedance was assessed by anthropometric measures. Body mass index (BMI) was calculated as body weight (kg) divided by height (m^2^). Subjects with 25.0 ≤ BMI ≤ 29.9 kg/m^2^ and with BMI ≥ 30 kg/m^2^ were classified in overweight and obese groups, respectively [21]. Term illiterate was defined as having little or no educational literacy. Low – educated subjects were considered as those completing< 5 years and/or 6–9 years of education. Also, subjects with 10–12 years and ≥ 13 years of education were classified in middle-educated and high-educated groups, respectively. We used wealth index as a proxy for economic status. Wealth index (WI) was generated by performing principal component analysis (PCA) on data related to durable goods, housing, and other amenities [22]. Information available on infrastructure (drinking water supply, sanitary facilities), housing conditions (e.g. the number of rooms, type of homeownership), possession of a variety of durable appliances (e.g. dishwasher, car, TV), and education levels in dataset was used to generate socio-economic status (SES) index for each participant. Study participants were divided into 5 SES groups from the lowest (1st) to the highest (5th) quintiles.

A national health interview Survey (NHIS) was used to evaluate smoking status. Those respondents who smoked ≥ 100 cigarettes in their lifetime were defined as smokers. Term “ex-smoker” referred to individuals who stopped smoking cigarettes and/or tobacco.

Term “former smoker” was intended to define those subjects who used to smoke, but now they don’t anymore [23]. Age of smoking initiation was determined based on the question: “How old were you when you first started to smoke fairly regularly?”, with a threshold of ≤ 18 being defined for early initiation.

Smoking cessation was some information self-reported by subjects who have stopped smoking at least for 4 weeks [24].

Following questions were used to assess self-reported exposure to SHS in childhood and at home/ work, respectively:

- 1a) Did any of your family members smoke tobacco products when you were a child?
- 1b) How many hours per day did you inhale secondhand smoke?
- 2) Did anyone smoke tobacco products at your home/ workplace when you were present there?

For question 2 assessing SHS exposures at work, those students (respondents) indicating “I do not have a job” were classified in a group with no exposure to SHS.

### Statistical analysis

For continuous variables, descriptive statistics were calculated and presented as mean ± SD (Standard deviation) and, for categorical variables, frequency (percentage) was expressed. Pearson’s chi-square test was employed to investigate correlation between 2 categorical variables.

Logistic, Poisson and linear regression models were used to examine determinates of smoking cessation expressed by odds rations (OR), of smoking intensity (the number of cigars per day) expressed by Incidence Risk Ratio (IRR), and of age of smoking initiation expressed by regression coefficients, respectively.

If alpha ≤ 0.3, then all variables from univariate analysis were included in multi-variate one. Backward stepwise elimination technique was used to remove those variables having no significant effects.

A two-sided alpha level of 0.05 was considered statistically significant. We excluded missing data (less than 1% of the whole data) for purposes of present study. All analyses were carried out using Stata software (version 14.1) (Stata Corp, College Station, TX, USA).

## Results

Ninety-six out of 10,182 individuals invited to participate in RaNCD refused to accept our invitation (participation rate = 99.06%). Smoking statuses of 51 subjects were not assessed. Out of 10,035 remaining participants, 4759 (47.42%) were male (Table 1).

**Table 1:** Demographic and clinical characteristics of participants in RaNCD by smoking status

***Variables***	***N (%)***	***Lifetime prevalence*** ***(95%CI)***	***Current*** ***(95%CI)***	***Ex_smoker*** ***(95%CI)***	Cessation Rate (%)
***Total***	***10,035 (100%)***	***2008 (20.0%)***	***1179 (11.7%)***	***829 (8.3%)***	*41.3*
**Gender**					
**Males**	4759 (47.42)	36.4 (35.1_37.7)	22.5 (21.4_23.7)	13.9 (12.9_14.8)	38.2
**Females**	5276 (52.58)	5.2 (4.6_5.8)	2.0 (1.7_2.4)	3.2 (2.7_3.7)	61.5
**Age group (year)**					
**35_45**	4418 (43.98)	14.4 (13.4_15.4)	10.4 (9.5_11.3)	4.0 (3.4_4.5)	27.8
**46_55**	3336 (33.26)	22.8 (21.4_24.2)	13.6 (12.4_14.7)	9.2 (8.3_10.2)	40.4
**56_65**	2281 (22.76)	26.8 (25.0_28.7)	11.7 (10.4_13.1)	15.1 (13.7_16.6)	56.3
**Marital status**					
**Married**	9050 (90.18)	21.0 (20.1_21.8)	12.3 (11.6_13.1)	8.7 (8.1_9.2)	41.4
**Single**	422 (4.21)	7.6 (5.3_10.7)	6.3 (4.3_9.3)	1.1 (0.5_0.2)	14.9
**Divorced/widow/widower**	563 (5.61)	13.3 (10.9_16.1)	6.4 (4.7_8.6)	6.9 (5.0_9.4)	51.9
**Education**					
**Illiterate**	2482 (24.73)	19.0 (22.06_24.54)	9.1 (8.2_9.9)	8.5 (7.7_9.4)	48.3
**≤ 5 years**	3832 (38.19)	16.9 (9.18_11.52)	14.4 (13.2_15.7)	8.1 (7.2_9.2)	36.0
**6_9 years**	1673 (16.67)	28.2 (6.721_10.11)	17.5 (15.3_19.9)	7.6 (6.1_9.3)	30.3
**10_12 years**	1267 (12.63)	22.8 (5.91_9.24)	13.0 (11.1_15.2)	8.6 (7.0_10.5)	39.8
**≥ 13 years**	781 (7.78)	16.3 (7.32_11.40)	8.6 (6.8_10.9)	7.4 (5.7_9.6)	46.3
**Residential areas**					
**Urban**	5943 (59.22)	18.6 (13.99_15.71)	10.6 (9.9_11.4)	7.9 (7.3_8.7)	42.7
**Rural**	4092 (40.78)	22.0 (15.82_18.09)	13.4 (12.3_14.4)	8.6 (7.8_9.5)	39.1
**Economic status**					
**Poorest**	1995 (20.00)	18.9 (17.10_20.38)	10.8 (9.5_12.4)	8.0 (6.9_9.3)	42.6
**2nd poorest**	1996 (20.01)	20.7 (15.39_18.82)	12.1 (10.8_13.6)	8.5 (7.3_9.8)	41.3
**Middle**	1995 (20.00)	20.6 (14.66_17.91)	12.2 (10.8_13.8)	8.4 (7.2_9.6)	40.8
**2nd richest**	2002 (20.07)	20.4 (13.19_16.34)	12.4 (11.0_13.9)	7.9 (6.8_9.2)	38.9
**Richest**	1988 (19.93)	19.3 (10.49_13.24)	10.9 (9.6_12.5)	8.2 (7.2_9.5)	42.9
**BMI**					
**18.4≤**	167 (1.68)	39.5 (3.60_11.87)	31.7 (25.3_38.9)	7.8 (4.7_12.5)	19.7
**18.5_24.9**	2742 (27.55)	25.5 (9.22_11.53)	16.7 (15.3_18.3)	8.7 (7.8_9.6)	34.3
**25.0–29.9**	4331 (43.51)	19.8 (13.99_16.14)	11.1 (10.1_12.1)	8.7 (7.9_9.4)	43.9
**30.0–34.9**	2130 (21.40)	13.7 (19.81_23.38)	6.5 (5.6_7.6)	7.1 (6.2_8.3)	52.2
**> 35**	584 (5.87)	11.6 (19.81_23.38)	5.1 (3.6_7.2)	6.5 (4.8_8.7)	56.0
**Childhood SHS**					
**No**	4919 (49.0)	13.8 (13.99_16.14)	8.4 (7.7_9.2)	5.4 (4.8_6.0)	39.1
**Yes**	5116 (51.0)	25.9 (19.81_23.38)	14.9 (13.9_15.9)	11.0 (10.1_11.8)	42.5
**Current SHS**					
**No**	4771 (47.40)	13.1 (13.99_16.14)	7.3 (6.6_8.0)	5.8 (5.1_6.5)	44.3
**Yes**	5294 (52.60)	26.2 (19.81_23.38)	15.7 (14.7_16.7)	10.4 (9.6_11.3)	39.8

Overall cessation rate was 41.3%. There was a difference in cessation rate between males and females. In addition, higher BMI, history of being divorced/ widow/ widower and living in urban areas were correlated with higher rates of smoking cessation (Table 1).

Univariate analysis showed a negative correlation between education levels and smoking cessation. Multivariate analysis showed that lower likelihood of smoking cessation was related to current exposure to SHS, higher smoking intensity and late smoking initiation. However, female gender, older age, higher education and higher BMI were related to increased likelihood of smoking cessation (Table 2).

**Table 2:** Determinants of Smoking cessation, Smoking intensity and Age of smoking initiation in crude and adjusted variables

Variable		Smoking cessation		Smoking intensity		Age of smoking initiation	
		Crude OR ***(95%CI)***	Adjusted OR ***(95%CI)***	Crude IRR ***(95%CI)***	Adjusted IRR ***(95%CI)***	Crude coefficient ***(95%CI)***	Adjusted coefficient ***(95%CI)***
**Gender**	**Male**	1	1	1	1	1	1
	**Female**	2.56 (1.97_3.32)	2.03 (1.47_2.80)	0.54 (0.52_0.56)	0.49 (0.47_0.52)	5.57 (4.53_6.62)	4.08 (2.94_5.22)
**Age group**	**35_45**	1	1	1	1	1	
	**46_55**	1.77 (1.41_2.22)	1.83 (1.44_2.33)	1.14 (1.11_1.18)	1.09 (1.06_1.12)	−0.5(−1.41_0.37)	
	**56_65**	3.36 (2.66_4.26)	3.58 (2.71_4.72)	1.06 (1.03_1.09)	1.10 (1.06_1.13)	2.17 (1.23_3.11)	
**Marital status**	**Married**	1		1	1	1	
	**Single**	0.26 (0.10_0.68)		0.77 (0.70_0.85)	0.87 (0.78_0.96)	0.68(−2.32_3.69)-	
	**Divorced/widow/widower**	1.54 (0.87_2.44)		0.81 (0.76_0.86)	1.35 (1.26_1.46)	3.86 (1.88_5.83)	
**Education**	**Illiterate**	1	1	1	1	1	1
	**≤ 5 years**	0.75 (0.59_0.95)	1.30 (0.59_0.95)	1.11 (1.08_1.14)	0.94 (0.91_0.98)	−2.96(−3.96_-1.95)	−1.59(− 2.64_-0.54)
	**6_9 years**	0.48 (0.37_0.63)	1.01 (0.37_0.63)	1.06 (1.03_1.10)	0.89 (10.86_0.93)	−2.70(−3.79_-1.62)	−1.02(− 2.20_-0.14)
	**10_12 years**	0.58 (0.43_0.78)	1.36 (0.43_0.78)	0.89 (0.86_0.93)	0.77 (0.73_0.80)	−2.55(− 3.79_-1.31)	−1.21(− 2.54_-0.11)
	**High school/ academic**	0.84 (0.57_1.25)	2.02 (0.57_1.25)	0.57 (0.53_0.60)	0.50 (0.46_0.53)	−2.23(−3.79_-0.59)	−1.83(− 3.57_-0.83)
**Place of resident**	**Urban**	1		1		1	
	**Rural**	0.86 (0.71_1.02)		1.02 (1.00_1.05)		0.75 (0.40_1.54)	
**Income status**	**Lowest**	1		1		1	
	**Lower**	0.94 (0.71_1.25)		1.14 (1.11_1.19)		0.09(−1.09_1.28)	
	**Middle**	0.92 (0.69_1.23)		1.15 (1.11_1.19)		−1.21(−2.40_-0.30)	
	**Higher**	0.86 (0.65_1.15)		0.99 (0.95_1.02)		−1.32(−2.51_-0.13)	
	**Highest**	1.02 (0.77_1.36)		0.93 (0.89_0.96)		−1.60(−2.81_-0.39)	
**BMI**	**18.4≤**	1	1	1	1	1	
	**18.5_24.9**	2.10 (1.12_3.94)	2.66 (1.39_5.12)	0.80 (0.76_0.85)	0.84 (0.79_0.89)	1.34(−0.82_3.50)	
	**25.0–29.9**	3.18 (1.71_5.93)	4.16 (2.17_7.95)	0.82 (0.77_0.86)	0.87 (0.82_0.93)	1.52(−0.62_3.67)	
	**30.0–34.9**	4.48 (2.34_8.58)	5.98 (3.04_11.7)	0.78 (0.73_0.83)	0.85 (0.80_0.91)	1.27(−1.00_3.56)	
	**> 35**	5.16 (2.38_11.1)	6.50 (2.87_14.6)	0.79 (0.73_0.86)	1.04 (0.95_1.13)	3.56 (0.70_6.42)	
**Childhood SHS**	**No**	1		1	1	1	1
	**Yes**	1.14 (0.94_1.38)		1.03 (1.09_1.14)	1.12 (1.00_1.26)	1.12 (1.09_1.14)	−0.94(−1.71_-0.18)
**Current SHS**	**No**	1	1	1	1	1	
	**Yes**	0.83 (0.68_1.00)	0.68 (0.52_0.87)	1.10 (1.11_1.17)	1.14 (1.06_1.41)	−0.38(−1.19_0.42)	
**Smoking intensity**	**Light smokers**	1	1	–	–	1	1
	**Medium smokers**	0.65 (0.50_0.84)	0.68 (0.51_0.91)			−3.54(−4.62_-2.45)	−2.91(−3.99_-1.84)
	**Heavy smokers**	0.55 (0.45_0.68)	0.57 (0.54_0.71)			−4.82(−5.64_ − 4.01)	-4.01(−4.85_-3.16)
**Age of smoking initiation**	**18≥**	1	1	1	1	–	–
	**> 19**	0.75 (0.58_0.86)	0.71 (0.58_0.86)	0.71 (0.70_0.73)	0.71 (0.70_0.73)		

**Fig. 1:**
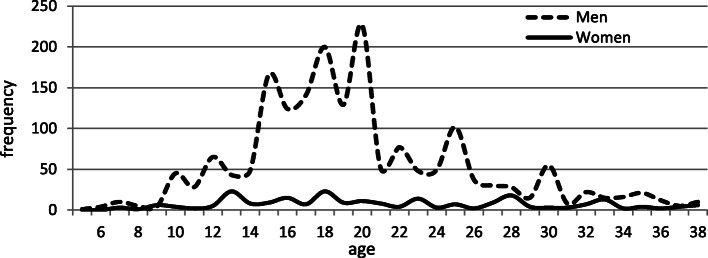
Age of smoking initiation in RaNCD by sex

Mean age of smoking initiation was 20.3 ± 7.8 yr for male and 25.19 ± 11 yr for female subjects. The highest proportion of smokers started to smoke at the age of 14–21, but this proportion decreased sharply after the age of 21 (Fig. 1).

After making adjustments of for variables, average age of smoking initiation was 4.08 yr (2.94–5.22) higher for women than that for men. The average age of smoking initiation increased by levels of BMI, but it was no longer different between BMI categories after making adjustments.

Although crude analysis indicated an association between living in rural areas and smoking initiation at older ages, that association disappeared after making adjustments for potential confounding factors. Also, heavy smokers started to smoke at younger ages in both uni- and multivariate models (− 4.01 yr).

Our multivariate analysis indicated correlations of increased smoking intensity with male gender, older age, being divorced/ widow/ widower, current and/or childhood exposure to SHS and lower levels of education while higher levels of physical activity and education were related to decreased risk of intense smoking (Table 2).

## Discussion

Smoking is one of the major health problems in all societies, having physical and mental/ psychological effects on individuals and endangering health of communities culturally, socially, economically and politically. In this study, smoking prevalence was assessed based on how people used 100 cigarettes in their lifetimes. Such a definition for the smoking status, according to Levy et al. [25], may have important implications for the smoking population size.

### Prevalence of smoking

Present study suggested a 20% prevalence of smoking, with male/ female subjects accounting for 36.4%/ 5.23%.

This rate of smoking prevalence in Ravansar is relatively higher than those in majority of other cities in Iran. For instance, mentioned rate is 19.2%/ 0.3% for men/ women in northern Iran [26], 22.7%/ 0.9% in Bandar Abbas (11.7%) [27], 24.1%/ 0.5% in Semnan (12.3%) [28, 29], 27.8%/ 10.7% in Hormozgan (19.5%) [30], 24.4%/ 14.6% in Tehran (20%) [31], 21.6%/ 0.4% in Bushehr (11.9%) [32], and 12.7%/ 2.0% in Birjand (7%) [33].

Furthermore, the rate of smoking prevalence in Iran is relatively higher in comparison with some countries including UK (16%) [34], Pakistan (14.2%) [35] and India (15.6%) [36]. On the other hand, this rate is lower in Iran compared to other countries including Vietnam (22.5%) [37], Turkey (30%) [38], Russia (63%) [39] and Egypt (40%) [40]. However, it should be noted that the rate of smoking prevalence in Iran is similar to that one reported on the US (20%) [41].

Perhaps, one reason why smoking is highly prevalent in this western city of Iran is environmental stress factors such as the eight-year Iran-Iraq war experienced by regional people. As a severe stressor, war entails a wide range of social, economic, cultural and personal outcomes affecting relationships among society members for many generations [42]. Iran-Iraq war was the second-longest one after Vietnam War in present century, lasting about 8 years and leaving hundreds of thousands of killed and injured people. However, casualties continued occurring although that war was over.

Psychological damages to the war survivors and their families inflicted irreparable damages on the community, with their adverse effects being still present [43]. Such effects are more pronounced in cities directly affected by war than in other parts of country.

Other reasons for higher rate of smoking prevalence in Ravansar include unemployment, poverty, lack of awareness, low levels of health literacy about dangerous consequences of smoking and false beliefs about benefits of smoking.

Results showed a relationship between smoking prevalence and gender, that is, smoking is more prevalent among males than females, which is in agreement with results obtained by Mehrabi et al. [17], Ebadi et al. [12], Agha Molaie and Zare [27], Van Minh et al. [37], Farshidi et al. [44], Javad et al. [45] and Borgan et al. [46].

Men are more likely to turn to smoking due to higher tendency toward risky behaviors and to encountering various stresses caused by occupational, family and social responsibilities, which may be the result of regional male dominance/ patriarchy. One key point revealed by our study is that rate of smoking prevalence is higher among females in Ravansar compared to other cities and regions of Iran such as northern Iran, Bandar Abbas, Semnan, Bushehr and Birjand. This requires much more investigations to be carried out on those women to identify reasons behind their tendency to smoke cigarettes. To this end, it is necessary to develop plans and programs specific to women since they can play an effective role in promoting health of their families and of society as a whole and in preventing potentially harmful effects of smoking on pregnancy and giving birth.

Results showed that smoking prevalence rate is lower in rural areas than that in urban ones, which are in agreement with results of studies by Sozanska et al. [47] and Nasir & Rehan [35], but which are in contrast to results obtained by Farshidi et al. [44], Mehrabi et al. [17] and Van Minh et al. [37] who demonstrated lower rate of smoking prevalence in urban areas. Moreover, results of study by Moradi et al. [48] indicated that there was no significant difference in the rate of smoking among residences of individuals.

Compared to people living in urban areas, those living in rural areas show healthier behaviors and are less exposed to stresses caused by traffic, crowd, and job responsibilities. In addition, rural people have a culturally negative attitude towards smoking.

### Smoking cessation

Based on results of this study, likelihood of quitting smoking is two times higher for women than for men (OR = 2.03; CI 95% (1.47–2.80)). Studies have shown that while smoking rate is lower among women than men, the latter quit cigarette smoking more successfully than the former [49, 50]. This is because, in comparison with men, women develop an increasingly negative mood, lose their ability to concentrate and suffer from loss of pleasure after they stop smoking, as a result of which they have lower chances to quit smoking. Considering that smoking has more side effects on women compared to men, they have more difficulties with quitting cigarettes than men do.

Our results showed a relationship between education levels and the number of trials to quit cigarettes. Subjects with higher levels of education did trials two times more than those with lower levels of education (OR = 2.02; CI 95% (0.57–1.25)). Such a result may be due to lower motivation and information (less use of professional and scientific sources while trying to quit smoking) as well as lower availability of sufficient resources and support for doing that. In line with these results, Zhuang et al. [51], Memar et al. [52], Reid et al. [53] and Lillard et al. [54] showed that lower-educated people had more difficulties with giving up smoking.

According to Nagelhout et al. [55], however, there was no relationship between education levels and giving up smoking.

Based on results of present study, people with higher BMI are 6.5 times more likely to quit smoking. People with high BMI seem to be more likely to die from a larger number of risk factors, so they get concerned about their health and try to reduce their unhealthy behaviors, one of which is smoking. Consequently, they are more motivated to quit cigarette smoking.

### Smoking intensity

Our results showed that smoking intensity is 1.35/0.87 times higher among divorced/ single individuals compared to married ones. Results of study by Ebadi et al. [12] showed that, given the marital status, likelihood of smoking is higher for divorced people compared to both single and married ones. By contrast, Mehrabi et al. [17] argued that, in comparison with married people, single individuals consumed lower number of cigarettes on the average. Married individuals were more concerned about their budgets and more likely to receive socio-psychological support acting as a protective shield against cigarette, alcohol and drug abuse compared to single and divorced people. It is unsuccessful marriage, negative mood and depression that lead divorced individuals to smoke more intensely than married/ single people do.

According to our results, childhood exposure to SHS increased likelihood of becoming a smoker by 1.14 times. Results obtained by Blacklock, Ghazu and Lorenz [56] indicated that children exposed to cigarette smoke were more likely to become smokers in teenhood and/or adulthood. Childhood exposure to cigarette smoke not only increases risk of smoking in later stages, but also causes a large number of complications such as low birth weight [57], sudden infant death syndrome (SIDS) [58], cognitive disorders [59], behavioral problems [59–61] and respiratory problems and asthma [62, 63]. Besides children, teenagers and adults exposed to cigarette smoke face a large number of problems [64].

Given the large number of problems and complications caused by the exposure to cigarette smoke, it is recommended that parents, especially pregnant mothers for the sake of their highly sensitive fetuses, quit cigarettes and/or avoid smoking at home or in public places.

To solve this problem, some effective steps include learning from experiences of other cities and countries with decreasing the rate of smoking successfully, implementing strict policies to reduce smoking, providing educational programs to inform community members of consequences and dangers of smoking, increasing the tax on cigarettes, preventing mass media from producing directly/indirectly advertisements for cigarettes and prohibiting smoking in public spaces.

It is suggested that results of present study be used by developers of the prevention programs designed to control tobacco.

Several factors may influence results of this study, including climatic and cultural differences, socio-economic conditions, health literacy, risk perceptions and people’s attitudes and beliefs about smoking.

According to our results, clearly, policies to control smoking have not been particularly successful at least in the study geographical location, Ravansar. Therefore, it can be recommended that such programs and plans be devised based on cultural and social conditions of this particular geographical location.

### Limitations and strengths

Our study had several limitations. We considered current number of cigarettes smoked by individuals, but we did not take information on previous years into account although it was available to researchers. We set an average variability for subjects’ smoking throughout their lifetimes. Finally, present research was done in one of Kurdish cities located at western Iran, therefore, caution needs to be taken in generalizing our results to other cultures and cities in Iran and in the world.

This study was the first population-based one with a large sample conducted in western Iran. In addition, subjects in the study sample were assessed by trained and experienced experts.

## Conclusions

Results of recruitment phase of a cohort study carried out in west of Iran showed that smoking prevalence was relatively high in the study area, with its rate being higher among married men with lower BMI who exposed to SHS and lived in rural areas. Failing to quit smoking successfully was common for current exposure to SHS, higher smoking intensity, later smoking initiation, male gender, younger age, lower education and underweight. Smoking intensity was lower among married people. Finally, childhood exposure to cigarette smoke resulted in an increased likelihood of becoming a smoker. Therefore, these issues should be considered in the process of planning and making policies to control smoking.

## Data Availability

All relevant raw data will be freely available to any scientists wishing to use them for non-commercial purposes without breaching confidentiality of participants.

## Abbreviations

RaNCD: Ravansar Non-Communicable Disease
SHS: Secondhand Smoke
PERSIAN: Prospective Epidemiological Research Studies in Iran
BMI: Body Mass Index
PCA: Principal Component Analysis
SES: Socio-economic Status Index
OR: Odds Ratios
IRR: Incidence Risk Ratios
